# MFGE8 is down‐regulated in cardiac fibrosis and attenuates endothelial‐mesenchymal transition through Smad2/3‐Snail signalling pathway

**DOI:** 10.1111/jcmm.15871

**Published:** 2020-09-17

**Authors:** Bo Wang, Zhuowang Ge, Yan Wu, Yafang Zha, Xuan Zhang, Yexiang Yan, Yuquan Xie

**Affiliations:** ^1^ Department of Cardiology Xinhua Hospital Affiliated to Shanghai Jiao Tong University School of Medicine Shanghai China; ^2^ Department of Nutriology Fudan University Shanghai Cancer Center Shanghai China; ^3^ Department of Cardiology Shanghai Tenth People's Hospital Chongming Branch Shanghai China; ^4^ Department of Cardiology Renji Hospital Affiliated to Shanghai Jiao Tong University School of Medicine Shanghai China

**Keywords:** cardiac fibrosis, EndMT, MFGE8, Smad2/3, TGF‐*β*1

## Abstract

Endothelial‐mesenchymal transition (EndMT) is a major source of transformed cardiac fibroblasts, which is reported to play a key role in cardiac fibrosis (CF), a pathogenesis of cardiovascular diseases such as heart failure, myocardial infarction and atrial fibrillation. Nonetheless, the specific mechanism underlying the progression of EndMT to CF is still largely unknown. In this study, we aimed to investigate the role of milk fat globule‐EGF factor 8 (MFGE8), a kind of soluble glycoprotein, in TGF‐*β*1‐induced EndMT. In animal experiments, the expression of MFGE8 was found down‐regulated in the left ventricle and aorta of rats after transverse aortic constriction (TAC) compared with the sham group, especially in endothelial cells (ECs). In in vitro cultured ECs, silencing *MFGE8* with small interfering RNA (siRNA) was found to promote the process of TGF‐*β*1‐induced EndMT, whereas administration of recombinant human MFGE8 (rh‐MFGE8) attenuated the process. Moreover, activated Smad2/3 signalling pathway after TGF‐*β*1 treatment and EndMT‐related transcription factors, such as Snail, Twist and Slug, was potentiated by *MFGE8* knock‐down but inhibited by rh‐MFGE8. In conclusion, our experiments indicate that MFGE8 might play a protective role in TGF‐*β*1‐induced EndMT and might be a potential therapeutic target for cardiac fibrosis.

## 1 INTRODUCTION

Heart failure (HF) is an end‐stage manifestation of many cardiac diseases leading to severe functional impairment and cardiac insufficiency. The basic pathogenesis of heart failure is thought to be associated with ventricular remodelling, which is characterized by excessive cardiac fibrosis (CF).^1^ Current studies indicate that myofibroblasts transformed from endothelial cells (ECs) through endothelial‐mesenchymal transition (EndMT) process are responsible for cardiac fibrosis.^2^, ^3^ During EndMT, ECs experience loss of epithelia polarity and endothelial markers (such as CD31 [PECAM1] and von Willebrand factor [vWF]) and increase of mesenchymal markers (such as Vimentin, alpha‐smooth muscle actin [*α*‐SMA], and fibroblast‐specific protein‐1 [FSP‐1]),^4^, ^5^ along with increased migration capacities and activated transcription factors (TF) such as Snail, Slug, Twist and Zeb1.^4^, ^5^ The role of EndMT in heart development,^6^, ^7^ pulmonary arterial hypertension,^5^ atherosclerosis,^8^ cardiomyopathy^9^ and CF^10^, ^11^ has always been a research hotspot. Meanwhile, targeting EndMT is generally thought to be a potential treatment for CF.

Milk fat globule‐EGF‐factor 8 (MFGE8) is a kind of lactadherin mainly secreted by mononuclear cells. This protein has been reported to be involved in various physiological functions and pathophysiological processes of the cardiovascular and cerebrovascular systems, including atherosclerosis,^12^ cardiac hypertrophy,^13^ angiogenesis,^14^ myocardial infarction (MI)^15^ and oxidative stress following subarachnoid haemorrhage.^16^ In previous studies, knockout of *Mfge8* in mice exacerbates pressure overload‐induced cardiac hypertrophy and leads to remarkable inflammatory responses and an enormous reduce in survival after MI.^15^ In addition, in recent years, a growing number of studies have emphasized the latent role of MFGE8 in organ fibrosis, such as blunting the degree of liver fibrosis in mice^17^ and decreasing tissue fibrosis in mice model with lung fibrosis.^18^ In our previous study, we found the protective role of MFGE8 in attenuating Ang‐II‐induced atrial fibrosis and atrial fibrillation through TGF‐β1‐Smad2/3 pathway.^19^ However, how MFGE8 functions and the inner molecular mechanisms between MFGE8 and EndMT remain to be explored.

In the present study, we identified MFGE8 as a vital regulator of cardiac fibrosis because it was found to be down‐regulated in rats with CF induced by transverse aortic constriction. In vitro knockout and administration experiments of ECs indicated that MFGE8 exerts protective action against transforming growth factor beta 1 (TGF‐*β*1)‐induced EndMT via the Smad2/3‐Snail signalling pathway. Data herein demonstrates the regulatory role of MFGE8 in EndMT and highlights the utility of MFGE8 as a diagnostic biomarker and a potential therapeutic target for CF.

## 2 MATERIALS AND METHODS

### 2.1 Animal studies and TAC model

Animal experiments were approved by the Animal Care and Use Committee of Xinhua Hospital and were performed under the guidance of the NIH Guide for the Care and Use of Laboratory Animals. 8‐week‐old male Sprague Dawley wild‐type rats (220‐240 g) were used and randomly assigned into different groups (n = 15 in each group). Cardiac fibrosis was induced by transverse aortic constriction (TAC). Briefly, operations were conducted after the rats were anesthetized intraperitoneally using pentobarbital sodium (50 mg/kg). After sternotomy, we tied a 27‐gauge needle along with the thoracic aorta tightly with a 7‐0 thread, then removed the needle and we could see a local narrow left in the aortic. Rats in the sham‐operated group went through the same operation procedure without undergoing ligation. Left ventricle (LV) and aorta tissues were obtained by the end of the 4th week after operations.

### 2.2 Histological analysis and immunofluorescence staining

LV and aorta tissues from the rats were fixed in 4% paraformaldehyde, embedded in paraffin and sliced into 4 µmol/L sections. Collagen of LV was stained by picrosirius red (PSR) to show the extent of interstitial fibrosis. Quantification of the fibrosis area was analysed using Image‐Pro Plus 6.0. Immunofluorescence staining of LV and aorta sections were performed using antibodies against MFGE8 (Abclonal, A12322), CD31 (Abclonal, A0378) and Vimentin (Abclonal, A2584) with a dilution factor of 1:200 according to methods described previously.^20^

### 2.3 Culture of cells

Human umbilical vein endothelial cells (HUVECs) were isolated from human umbilical vein as previously described.^21^ Human microvascular endothelial cells (HMECs) were purchased from Lonza. All these cells were cultured with EGM‐2 BulletKit (Lonza, CC‐3124) in a humid environment at 37°C, 5% CO2. Recombinant human TGF‐*β*1 (10 ng/mL, Peprotech) was added to stimulate the cells for 48 hours to induce EndMT, and recombinant human MFGE8 (rh‐MFGE8, 500 ng/mL, R&D) resolved in sterile phosphate buffered solution (PBS) was used.

### 2.4 RNA interference

Chemically synthesized small interfering RNAs (siRNAs) targeting human *MFGE8* were purchased from Dharmacon (Lafayette, CO). According to the manufacturer's instructions, cultured cells were transiently transfected with 50 nmol/L siRNA using Lipofectamine™2000 (Life Technologies). The specific siRNA that provided the most efficient inhibition (resulted in a 75%‐80% decrease in mRNA and protein levels compared with negative control siRNA [si‐NC]) was used for the following experiments. The targeting sequences of the siRNAs are (a) GGAACATTGCCAACTCACA; (b) GGACACGAATTCGATTTCA; and (c) GTGGGTAACTGGAACAAAA.

### 2.5 Quantitative real‐time PCR

Total RNA isolated from cultured cells using TRIzol reagent (Invitrogen, 15596018) was transcribed into complementary DNA (cDNA) using PrimeScript RT kit (Takara, RR036A). Quantitative real‐time polymerase chain reaction (qRT‐PCR) was performed using SYBR Green (Takara, RR420A) in 20 μL reaction volumes. Results were normalized to the expression of glyceraldehyde‐3‐phosphate dehydrogenase (GAPDH). Primer pairs are listed in Table 1.

**Table 1:** Primers sequences used for qRT‐PCR

	Gene	Forward primer	Reverse primer
Human	MFGE8	CTCGTCTGTGCGTGTGACCTTC	CTGCGTCACCACACCTGTTACC
Human	CD31	CGTCAAGCCTCAGCACCAGATG	GCACTCCTTCCACCAACACCTG
Human	Vimentin	TTGCCGTTGAAGCTGCTAACTACC	AATCCTGCTCTCCTCGCCTTCC
Human	ACTA2	CAGGTCATCACCATCGGCAACG	GATGCTGTTGTAGGTGGTCTCGTG
Human	FSP1	TGAGCAACTTGGACAGCAACAGG	TTACACATCATGGCGATGCAGGAC
Human	Snail	AATCCAGAGTTTACCTTCCAGC	GAAGTAGAGGAGAAGGACGAAG
Human	Twist	GTACATCGACTTCCTCTACCAG	CATCCTCCAGACCGAGAAG
Human	Slug	CTGTGACAAGGAATATGTGAGC	CTAATGTGTCCTTGAAGCAACC
Human	GAPDH	CCAGAACATCATCCCTGCCT	CCTGCTTCACCACCTTCTTG

### 2.6 Western blot analysis

Proteins from the cultured cells and LV were extracted by RIPA solution (Beyotime, P0013B) with a mixture of protease and phosphatase inhibitor (Thermo Scientific, 78441), and the concentrations were quantified using BCA protein estimation kit (Thermo Scientific, 23225). Equal quantities of the protein samples (20‐40 μg) were separated via SDS‐PAGE and electro‐transferred to 0.22 μm polyvinylidene fluoride (PVDF) membranes. After blockage, membranes were incubated overnight at 4°C with the respective primary antibodies against MFGE8 (Abclonal, A12322), CD31 (Abclonal, A0378), Vimentin (Abclonal, A2584), *α*‐SMA (Abclonal, A7248), FSP1 (Abcam, ab124805), Snail (Abcam, ab216347), Twist (Abcam, ab175430), Slug (Abcam, ab51772), p‐Smad2/3 (Abclonal, AP0548), Smad2/3 (Abclonal, A7536), TGF‐βRI (Abclonal, A11934), Smad4 (Abclonal, A19116) and GAPDH (Abclonal, AC027) of the dilution factor 1:1000. Then, after thoroughly washing with TBS‐T, respective secondary antibody conjugated with HRP (1:1000) was incubated for 1 hour at room temperature, and signals were visualized using ECL reagents on Tanon 5200. Immunoblots were quantified by densitometry using ImageJ software.

### 2.7 Cellular immunofluorescence

Cells cultured on cover slips were fixed in 4% paraformaldehyde for 15 minutes after stimulation. Following permeabilization with 0.1% Triton X‐100 for 20 minutes and blockage for 40 minutes, the cells were incubated overnight with primary antibodies against CD31 (Abclonal, A0378) and/or *α*‐SMA (Abclonal, A7248) at 4°C. Secondary antibody conjugated with Alexa Fluor 488 and/or Alexa Fluor 594, and DAPI were used for visualization.

### 2.8 Scratch assay

HUVECs were seeded in 6‐well plates at a density of 5.0 × 105 cells/mL. When cells reached the confluency of 90%, cell monolayer was scratched using a sterile 200 μL pipette tip and gently washed twice with PBS. Different marks were drawn at the bottom of the wells to ensure that the same visual fields were assayed. Images were taken at 0, 6, 12 and 24 hours post‐scratching and Image‐Pro Plus 6.0 was used to measure the distance of migration.

HUVECs were seeded in 6‐well plates at a density of 5.0 × 105 cells/mL. When cells reached the confluency of 90%, cell monolayer was scratched using a sterile 200 μL pipette tip and gently washed twice with PBS. Different marks were drawn at the bottom of the wells to ensure that the same visual fields were assayed. Images were taken at 0, 6, 12 and 24 hours post‐scratching and Image‐Pro Plus 6.0 was used to measure the distance of migration.

### 2.9 Transwell migration assay

Samples containing 1 × 105 cells were resuspended in serum‐free medium and added into the upper layer of Transwell Chambers (8 μm pore size, Corning, 3413). After incubated for 24 hours with complete medium in the lower layer, the chambers were washed, fixed and stained with crystal violet. Numbers of cells in five randomly selected fields were counted to assess cell migration.

### 2.10 Angiogenesis assay

Matrigel (Corning, 354248) was added into 96‐well plates and allowed to facilitate solidification at 37°C for 1 hour. Then, suspension of 15 000 cells with different stimuli was gently pipetted into each well and incubated for 18 hours in a standard environment. Tube formation was observed by microscopy.

### 2.11 Statistical analysis

All data were presented as means ± SEM and analysed by SPSS 19.0 statistical software and GraphPad Prism 6.0. Statistical analysis was performed using two‐tailed Student's *t* test and one‐way ANOVA. Differences were considered statistically significant at *P* < .05.

## 3 RESULTS

### 3.1 MFGE8 expression is decreased in endothelial cells of rats with cardiac fibrosis

**Figure 1:**
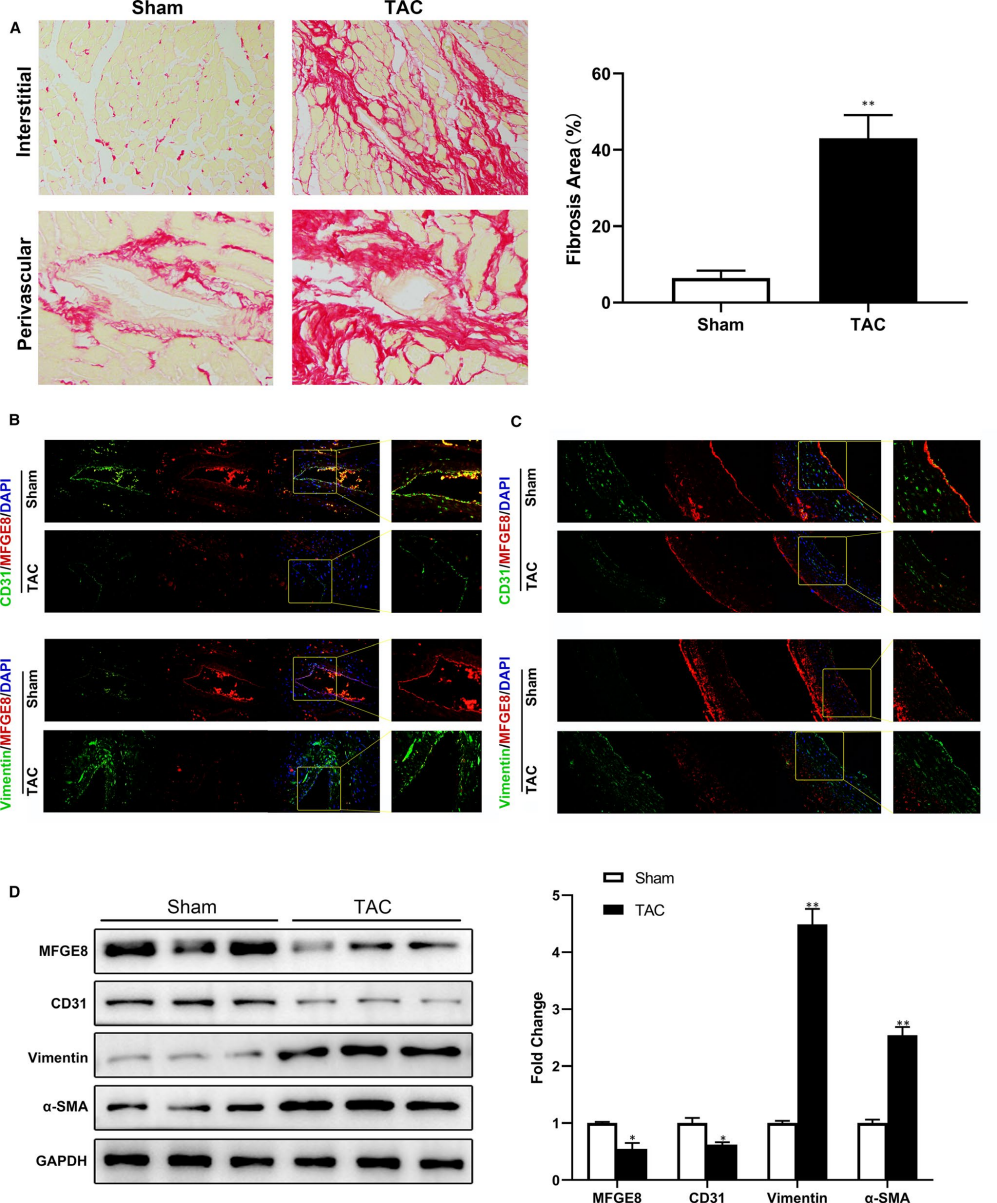
MFGE8 expression was down‐regulated in rats with TAC‐induced cardiac fibrosis. A, Representative picrosirius red (PSR) staining and quantification of cardiac fibrosis (n = 6 in each group; original magnification, ×200). B, Double‐immunofluorescence images of the left ventricle (LV) obtained from rats by the end of the 4th week after operations. (n = 6 in each group; up: green, CD31; red, MFGE8; blue, DAPI; down: green, Vimentin; red, MFGE8; blue, DAPI; original magnification, ×400). C, Double‐immunofluorescence images of the aortic tissues from rats (n = 6 in each group; up: green, CD31; red, MFGE8; blue, DAPI; down: green, Vimentin; red, MFGE8; blue, DAPI; original magnification, ×400). D, MFGE8 and EndMT‐related protein levels in LV tissues by Western blot analysis and quantitation. n = 6 in each group. Data are presented as mean ± SEM. **P < .01 vs sham group (one‐way ANOVA followed by Student's *t* test)

Previous studies indicate that MFGE8 is under‐express in cardiovascular and cerebrovascular diseases. To expound the role of MFGE8 in cardiac fibrosis, we performed TAC in rats and analysed MFGE8 expression in the left ventricle and aorta. The positive area of PSR staining demonstrated cardiac fibrosis in TAC‐treated rats (Figure 1A). In order to investigate whether EndMT occurred in rats with cardiac fibrosis, immunofluorescence staining was used to detect the expression of endothelial cell markers CD31, mesenchymal cell markers Vimentin and MFGE8. As is shown, CD31 and MFGE8 were highly expressed in vascular endothelial cells of left ventricle and aorta in the sham‐operated group. However, in TAC group, the fluorescence intensity of CD31 and MFGE8 in ECs decreased, while the fluorescence intensity of Vimentin increased, suggesting that EndMT occurred in rats with cardiac fibrosis. In both LV and aorta, MFGE8 is prominently co‐located with CD31 in ECs, suggesting that MFGE8 may be involved in EndMT (Figure 1B,C). Western blot experiment further confirmed the decreased MFGE8 expression in ventricular tissues of rats (Figure 1D). These data indicated that vascular endothelial cells of rats with cardiac fibrosis undergo endothelial‐mesenchymal transformation, and MFGE8 might have a hand in this pathological process.

### 3.2 TGF‐β1 induced endothelial‐mesenchymal transition in ECs cultured in vitro

**Figure 2:**
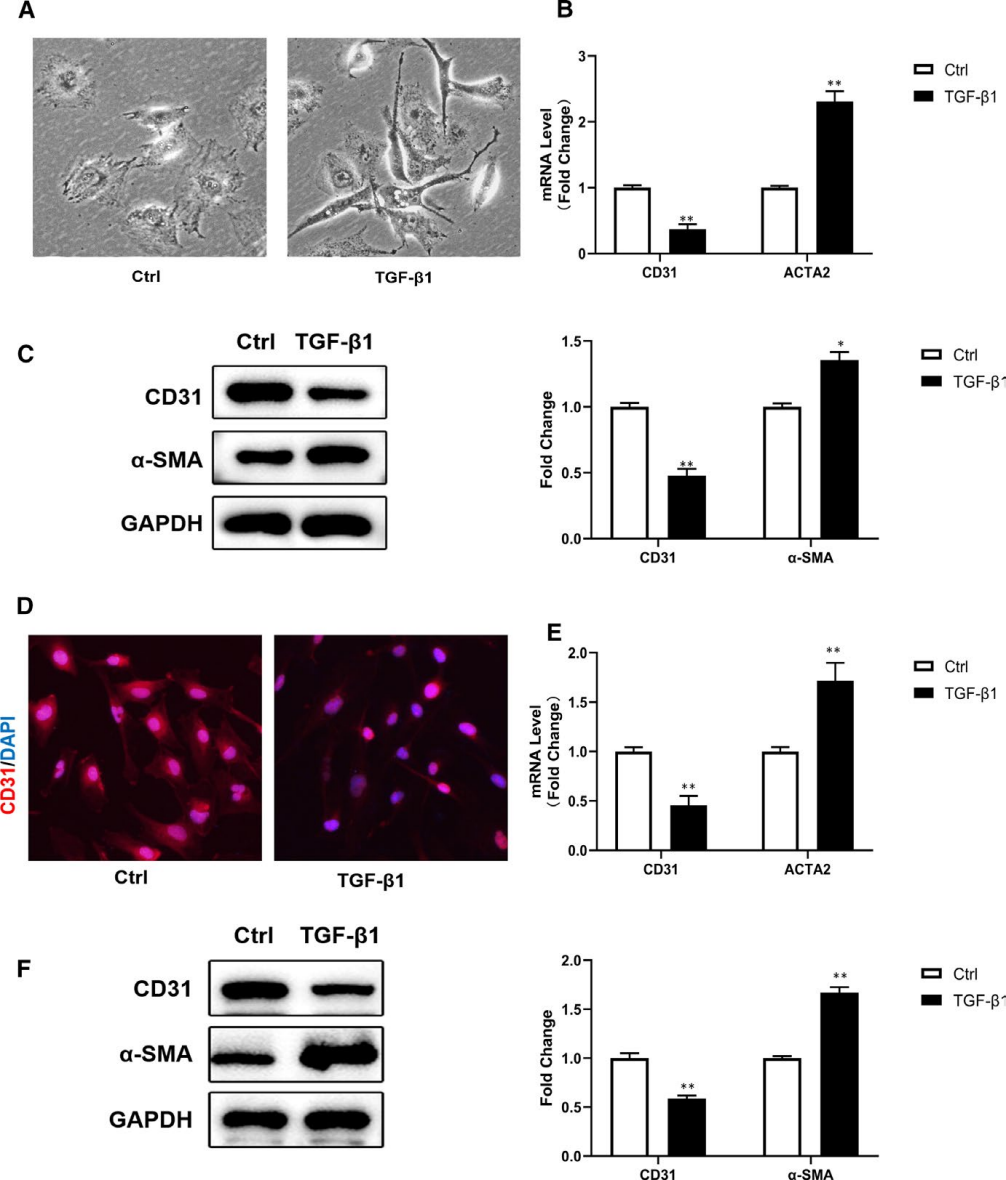
TGF‐*β*1 could induce EndMT in HUVECs and HMECs cultured in vitro. A, Morphological changes in HUVECs observed under light microscope. (n = 4 in each group; original magnification, ×400). B, D, Estimation of relative levels of mRNA expression of endothelial and mesenchymal markers in HUVECs (B) and HMECs (D) by qRT‐PCR. C, F, EndMT‐related protein levels in HUVECs(C) and HMECs (F) by western blot. D, Cellular immunofluorescence of HMECs for the expression level of CD31 (red) and nuclei (blue). (n = 4 in each group; original magnification, ×400). Data are presented as mean ± SEM. *P < .05, **P < .01 vs corresponding control (Ctrl) group (one‐way ANOVA followed by Student's *t* test)

To establish an EndMT cell model in vitro, we stimulated cultured HUVECs and HMECs with TGF‐*β*1 (10 ng/mL) for 48 hours. Obvious variations in cell morphology were observed post‐stimulation, changing from a cobblestone shape of endothelial cells to a shape of spindle‐like fibroblasts (Figure 2A,D). The results of qRT‐PCR authenticated the levels of the corresponding messenger RNA (mRNA). In the presence of TGF*β*1, the level of CD31 mRNA was greatly reduced while ACTA2 mRNA levels were increased (Figure 2B,E). Western blot analysis provided further evidence that ECs experiencing EndMT lost the molecular marker CD31 and began to express molecular markers of mesenchymal or myofibroblast, such as *α*‐SMA (Figure 2C,F). The double‐immunofluorescence images of HMECs provided evidence not only for changes in shapes, but also for the weakened CD31 signal (Figure 2D). All these results indicated that ECs experienced EndMT under the stimulation of TGF‐*β*1.

### 3.3 Knock‐down of MFGE8 with siRNA promoted EndMT in HMECs

**Figure 3:**
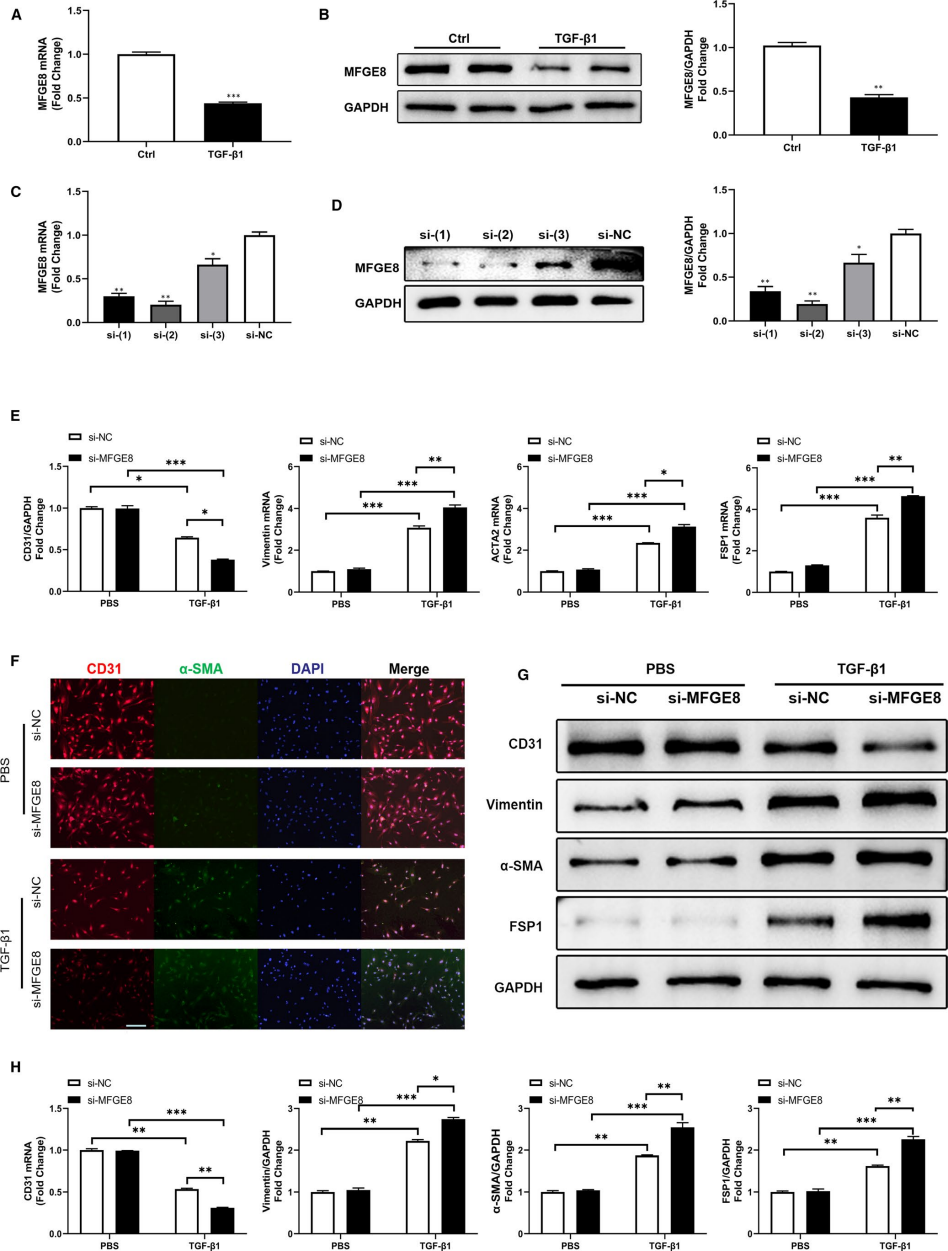
siRNA‐mediated silencing of MFGE8 promoted EndMT induced by TGF‐*β*1 in HMECs. A, B, Decreased level of MFGE8 expression in HMECs upon stimulation by TGF‐*β*1 assessed by qRT‐PCR (A) and Western blot (B). n = 4 in each group. C, D, Knock‐down efficiency of three MFGE8 siRNAs with different interference sequences verified at mRNA (C) and protein (D) levels. n = 4 in each group. E, Relative mRNA levels of endothelial and mesenchymal markers by qRT‐PCR. F, Cellular immunofluorescence of HMECs for the expression of CD31 (red), *α*‐SMA (green), and nuclei (blue) (n = 6 in each group; original magnification, ×200). G, H, EndMT‐related protein levels assessed by Western blot (G) and statistical quantitation (H). n = 4 in each group. Data are presented as mean ± SEM. *P < .05, **P < .01 and ***P < .001 compared with corresponding control (si‐NC or PBS) group or between the 2 indicated groups (one‐way ANOVA followed by Student's *t* test)

As expected, the mRNA and protein levels of MFGE8 was down‐regulated approximately to 50% in HMECs after TGF‐*β*1 stimulation (Figure 3A,B). Next, to explore the role of MFGE8 in TGF‐*β*1‐induced EndMT, three *MFGE8*‐specific siRNAs were transfected into HMECs for 24 hours to down‐regulate MFGE8, and their knock‐down efficiency was corroborated by qRT‐PCR (Figure 3C) and Western blot (Figure 3D) post‐transfection. As we found, si‐(2) had the most significant knock‐down efficiency, which could reduce the expression of MFGE8 mRNA and protein by 70%‐80%, and was used in the subsequent experiments. At mRNA (Figure 3E) and protein levels (Figure 3G,H), *MFGE8* knock‐down decreased the ECs marker CD31 and in contrast increased the TGF‐*β*1‐induced expression of the mesenchymal markers Vimentin, ACTA2 (*α*‐SMA) and FSP1. Same results of changes in markers were also observed by double‐immunofluorescence. In addition, from the immunofluorescence images, it could be seen that *MFGE8* knock‐down HMECs have a tendency to transform into spindle‐like fibroblasts (Figure 3F). Experiments above demonstrated that *MFGE8* silencing could promote EndMT process induced by TGF‐*β*1 in HMECs.

### 3.4 Knock‐down of MFGE8 by siRNA exacerbates EndMT in HUVECs

**Figure 4:**
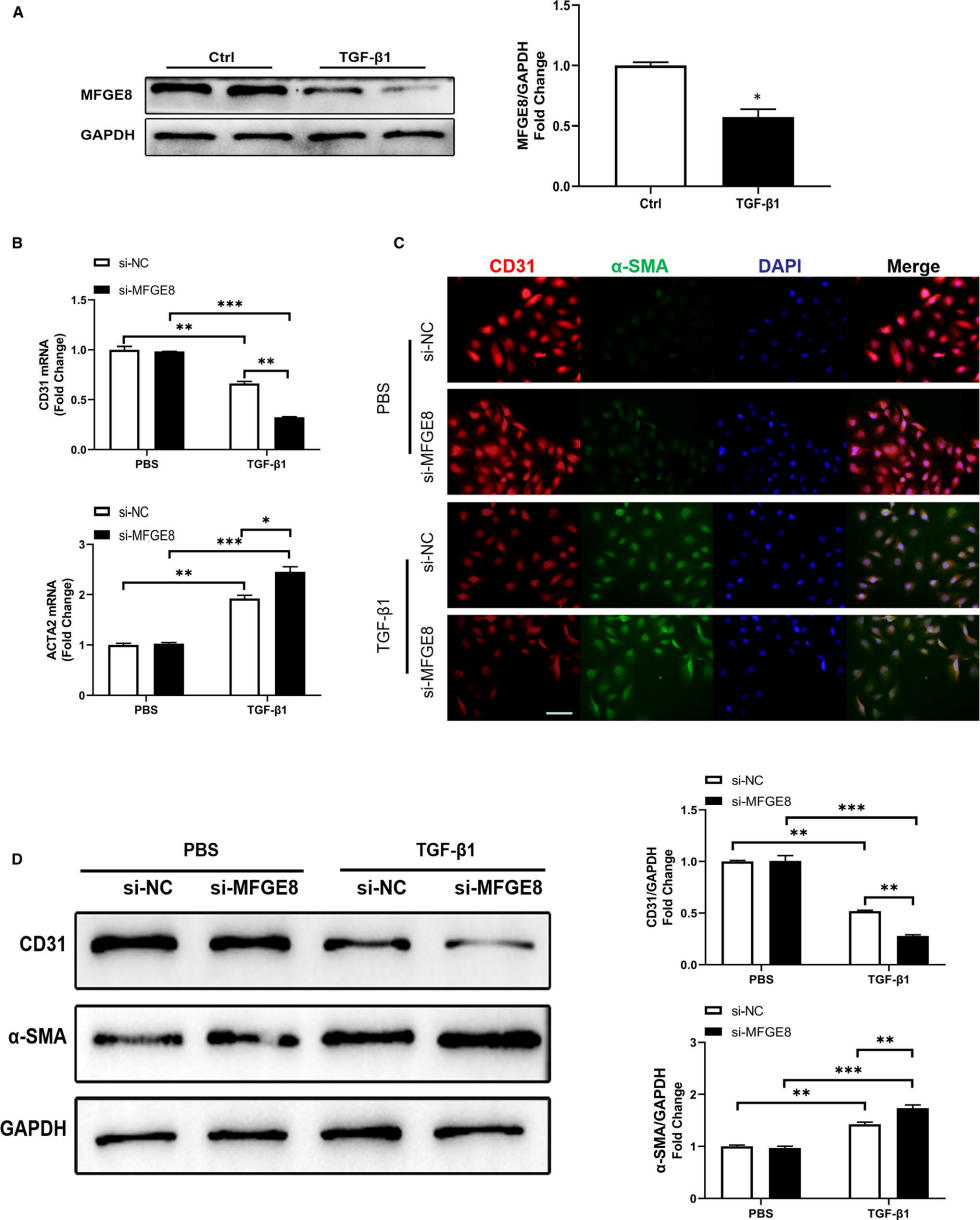
siRNA‐mediated knock‐down of *MFGE8* gave impetus to EndMT induced by TGF‐*β*1 in HUVECs. A, Decreased protein level of MFGE8 in TGF‐*β*1‐stimulated HUVECs by Western blot. n = 4 in each group. B, Related mRNA levels of endothelial and mesenchymal markers by qRT‐PCR. C, Cellular immunofluorescence of HUVECs for the expression of CD31 (red), *α*‐SMA (green) and nuclei (blue) (n = 6 in each group; original magnification, ×200). D, Levels of EndMT‐associated proteins markers assessed by Western blot and the corresponding quantitative analysis. n = 4 in each group. Data are presented as mean ± SEM. *P < .05, **P < .01 and ***P < .001 vs corresponding control (Ctrl or PBS) group or between the 2 indicated groups (one‐way ANOVA followed by Student's *t* test)

To further confirm the effect of MFGE8 on EndMT in different ECs, we analysed its expression at protein levels in HUVECs and found a 40% decrease in TGF‐*β*1‐treated group (Figure 4A). Then, si‐MFGE8 was transfected into HUVECs. Compared with si‐NC, si‐MFGE8 remarkably strengthened the effect of TGF‐*β*1 in inducing EndMT for the decrease of CD31 and increase of *α*‐SMA in both mRNA (Figure 4B) and protein levels (Figure 4D). Moreover, immunofluorescence images provided intuitive and similar results (Figure 4C). Generally, the data clarified that knock‐down of *MFGE8* gave impetus to EndMT induced by TFG‐*β*1 in HUVECs.

### 3.5 MFGE8 silence inhibits angiogenesis and promotes cell migration in HUVECs

**Figure 5:**
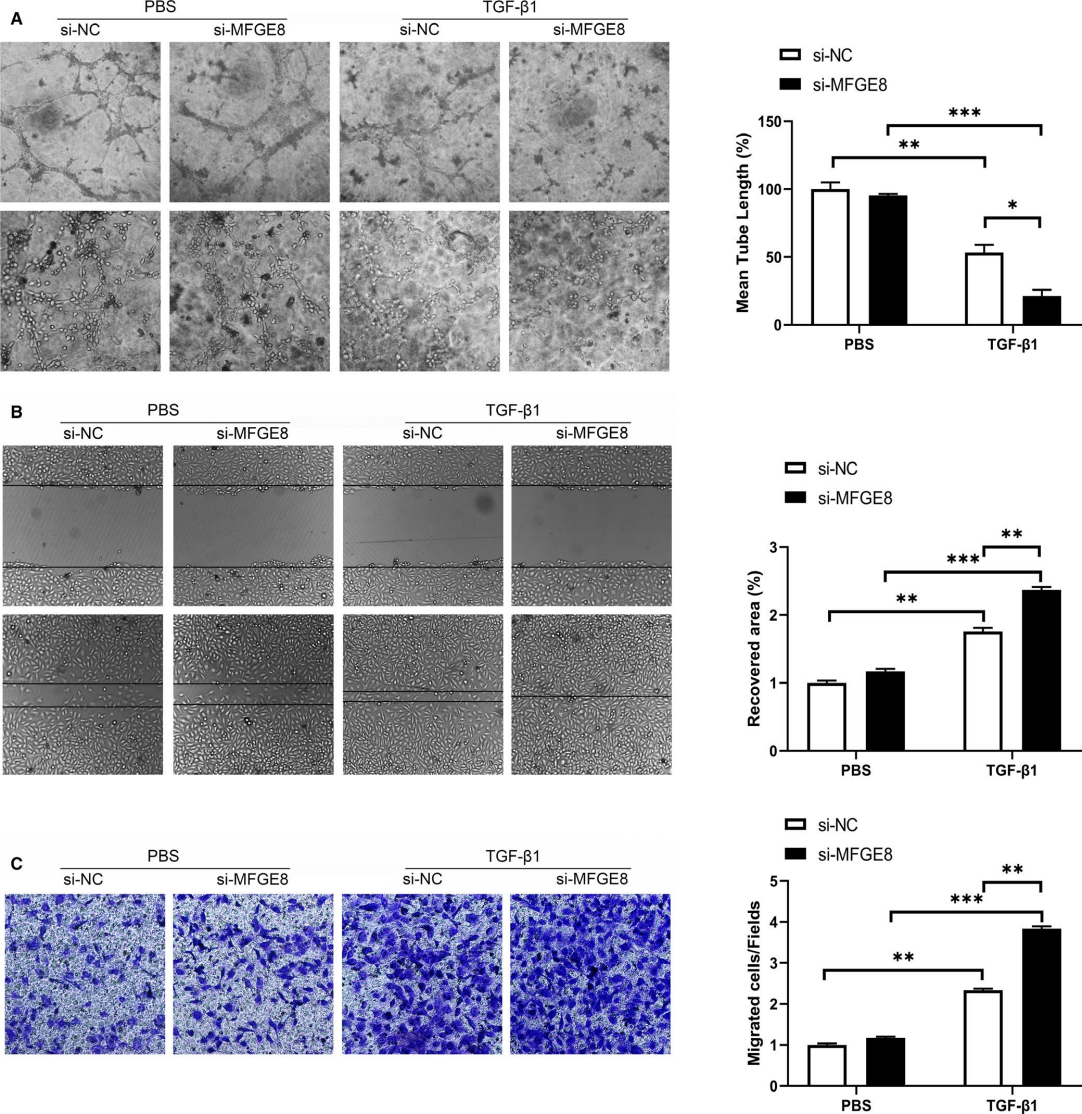
*MFGE8* silence inhibited angiogenesis and promoted cell migration in HUVECs. A, Representative images of angiogenesis assay in HUVECs under different stimulations (n = 4 in each group; original magnification, up: ×100; down: ×200). B, Evaluation of cell migration ability by scratch assay (n = 4 in each group; original magnification, ×100). C, Influence of *MFGE8* knock‐down on cell migration assessed by Transwell assay (n = 4 in each group; original magnification, ×200). Data are presented as mean ± SEM. *P < .05, **P < .01 and ***P < .001 vs PBS group or between the 2 indicated groups (one‐way ANOVA followed by Student's *t* test)

Subsequently, we conducted angiogenesis assay to evaluate the influence of MFGE8 on angiogenesis, which is reported to be a characteristic of partial EndMT. Our data revealed that si‐MFGE8 transfection led to a great decrease of angiogenesis ability compared with the group with TFG‐*β*1 treatment alone (Figure 5A). Furthermore, results of the scratching assay (Figure 5B) and transwell assay (Figure 5C) proved the enhanced endothelial cell migration caused by *MFGE8* silence in TFG‐*β*1 group. These experiments confirmed that silence of *MFGE8* further promoted the development of EndMT in the pathological environment induced by TGF‐*β*1.

### 3.6 rh‐MFGE8 reverses EndMT induced by TGF‐β1 in HUVECs

**Figure 6:**
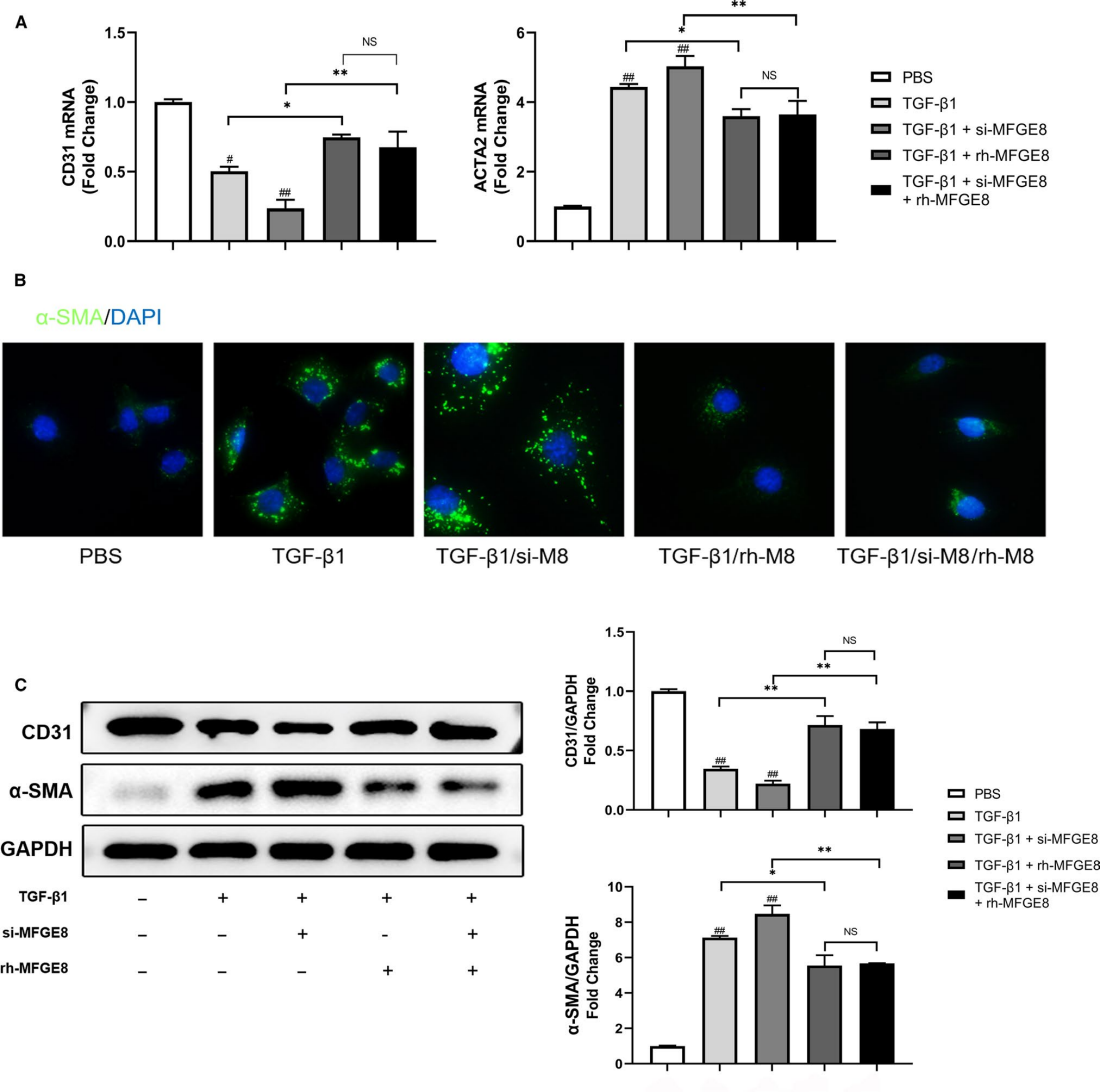
rh‐MFGE8 reverses EndMT induced by TGF‐*β*1 in HUVECs. A, mRNA levels of CD31 and ACTA2 by qRT‐PCR. n = 4 in each group. B, Cellular immunofluorescence of HUVECs for the expression level of *α*‐SMA (green) (nuclei (blue); n = 6 in each group; original magnification, ×400). C, Protein levels of CD31 and *α*‐SMA by Western blot. n = 4 in each group. Data are presented as mean ± SEM. *P < .05 and **P < .01 between the 2 indicated groups. #*P* < .05 and ##*P* < .01 vs corresponding PBS group (one‐way ANOVA followed by Student's *t* test)

In order to investigate whether exogenous MFGE8 could play a protective role in EndMT, we introduced recombinant human MFGE8 (rh‐MFGE8) into HUVECs. As is shown in Figure 6, administration of rh‐MFGE8 attenuated the EndMT caused by TGF‐*β*1 dramatically, even under the existence of si‐MFGE8. The expression of CD31 mRNA increased on the baselines of TFG‐*β*1 group and TFG‐*β*1 + si‐MFGE8 group, while the expression of ACTA2 mRNA decreased (Figure 6A). Changes of protein expression were consistent with the results of qRT‐PCR (Figure C). Besides, decreased *α*‐SMA expression in HUVECs cultured with rh‐MFGE8 was observed by dual‐immunofluorescence staining (Figure 6B). The results highlighted that endogenous rh‐MFGE8 could attenuate the process of TGF‐*β*1‐induced EndMT in vitro.

### 3.7 MFGE8 regulates EndMT induced by TGF‐β1 through Smad2/3 signalling pathway and influences the expression of EndMT‐related transcription factors

**Figure 7:**
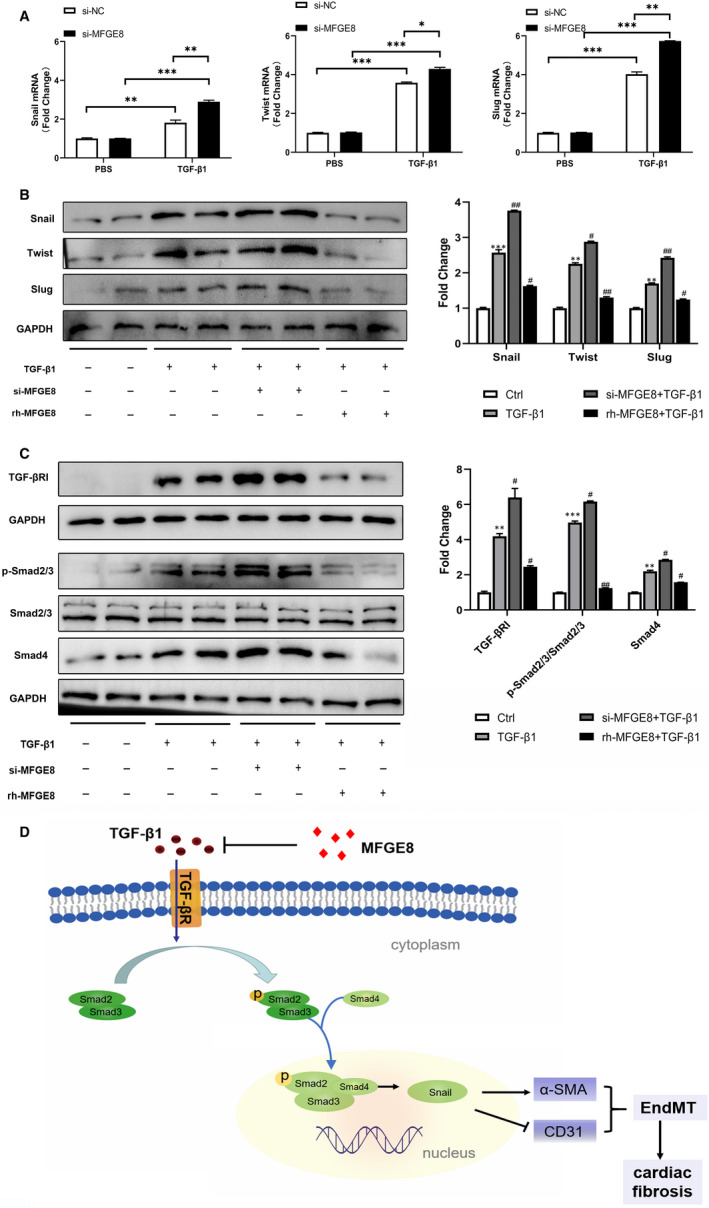
MFGE8 regulates EndMT induced by TGF‐*β*1 through Smad2/3 signalling pathway and influences the expression of EndMT‐related transcription factors. A, B, Expression levels of EndMT‐associated transcription factors in HUVECs by qRT‐PCR (A) and Western blot (B). n = 4 in each group. C, Typical blot results and quantitative analysis of TGF‐βRI and Smad2/3 pathway in different treatment groups in HUVECs. n = 4 in each group. D, Schematic diagram of the hypothesis. Arrows or lines indicate the activation or inhibition relationship between the two molecules. Data are presented as mean ± SEM. *P < .05, **P < .01, ***P < .001 and NS indicate no significance vs corresponding control (Ctrl or PBS) group or between the 2 indicated groups. #*P* < .05 and ##*P* < .01 vs corresponding TGF‐*β*1 group (one‐way ANOVA followed by Student's *t* test)

Our next aim was to elucidate the underlying mechanism of MFGE8‐mediated regulation of TGF‐*β*1‐induced EndMT in vitro. Previous studies have confirmed that transcription factors Snail, Twist and Slug are closely related to EndMT.^22^ To mimic the knock‐down and overexpress status of MFGE8 in ECs in vitro, we treated the cultured cells with si‐Mfge8 or rh‐MFGE8 for 24 hours. According to the results of qRT‐PCR analysis, the mRNA expressions of Snail, Twist and Slug were increased remarkably in TGF‐*β*1‐treated cells vs PBS‐treated cells, and the cells treated with si‐MFGE8 showed the most significant increase (Figure 7A). In Western blot analysis, visualization results and quantitative analysis showed that the combination of TGF‐*β*1 and si‐MFGE8 synergistically induced the expression of these EndMT‐related transcription factors while administration of rh‐MFGE8 attenuated the increasing trend (Figure 7B). Furthermore, Smad2/3 are known to be canonical transcription factors downstream of TGF‐*β* pathway. TGF‐*β* binds to type II receptor (TGF‐*β*RII) and recruits TGF‐*β* receptor type I (TGF‐*β*RI) in the plasma membrane. Then, Smad2/3 was phosphorylated and binds to the co‐activator Smad4 to transmit signals to the nucleus.^21^ According to previous studies, Smad4 plays a common‐partner role in the Smads family.^23^ Once activation, receptor‐regulated Smads (Smad2, 3) recruits Smad4 to form heteromeric complexes and accumulate in the nucleus to regulate the transcription of various genes.^24^ Compared with cells in the control groups, the expression of TGF‐*β*RI, phosphorylated Smad2/3 and Smad4 was elevated upon stimulation with TGF‐*β*1, indicating the activation of the signalling pathway. In si‐MFGE8‐treated HUVECs, the expression of TGF‐*β*RI was more strongly induced, and conversely, rh‐MFGE8 reversed the increased expression induced by TGF‐β1 (Figure 7C) and the expression of phosphorylation of Smad2/3 and Smad4 showed a parallel change. These results were also confirmed in HMECs (Figure S1). Taken together, these observations indicated that MFGE8 regulated EndMT of endothelial cells via TGF‐*β*RI, Smad2/3 signalling pathway and adjusted the expression of related transcription factors.

## 4 DISCUSSION

Cardiac fibrosis is a critical contributor to the progression of heart failure. It is due to excessive deposition of extracellular matrix caused by activation and accumulation of cardiac fibroblasts and results in a shrinkage of microvascular system and destruction of normal myocardial structure.^25^ During development, fibroblasts derived from ECs undergo epithelial‐mesenchymal transition (EMT),^26^ while in pressure overload‐induced cardiac lesions, they are mainly derive from pathologically induced EndMT.^10^ Studies have verified that a variety of environmental factors including hypoxia, radiation, high blood glucose levels, inflammation and TGF‐*β* can trigger EndMT.^27^, ^28^, ^29^, ^30^, ^31^ In our previous study, high glucose could induce EndMT in HUVECs through Smad2/3 pathway.^32^ Compelling evidence suggests that Smad proteins are important in cardiovascular differentiation and cardiac fibrosis. When the level of TGF‐*β* increases, TGF‐*β* receptor emits signal to recruit and phosphorylate Smad2/3. Upon phosphorylation, Smad3 plays a pivotal role in promoting signal cascade conduction by assembling a complex with Smad4.^23^ These Smads complexes translocate to the nucleus and interact with Snail, Slug, Twist and Zeb1 transcription factors. As a result, endothelial gene expression is inhibited and mesenchymal gene expression is induced, thus promoting EndMT. Among the 3 isoforms of TGF‐*β*, TGF‐*β*1 is the most commonly studied in the context of pathological EndMT, while TGF‐*β*2 is the most important in physiological process.^33^ So we used TGF‐*β*1 to induce EndMT in ECs in the present study. During this cellular transition process, ECs undergo molecular and structural rearrangements. They lose their endothelial characteristics and cell adhesion, decrease expression of surface EC markers such as CD31 and vWF, and obtain high migration potential and fibroblast‐like phenotype, which is corresponding with our results in Figure 2.

From another perspective, fibrotic disease can be caused by exaggerated apoptosis and inflammation and is characterized by collagen‐rich matrix replacing normal tissue structure and organ dysfunction.^34^, ^35^, ^36^ MFGE8 (also known as lactadherin) is a soluble glycoprotein which plays a negative role in regulating inflammation and autoimmunity by facilitating apoptotic cell clearance.^37^ In recent years, the anti‐fibrosis function of MFGE8 has been widely studied. Atabai et al confirmed that *Mfge8* ^−/−^ mice exhibit a severe pulmonary fibrosis phenotype after bleomycin treatment for the discoid domain of MFGE8 was sufficient to bind and internalize collagen and promoted the removal of collagen accumulated in tissues.^18^ In another study, MFGE8 secreted by mesenchymal stem cells (MSCs) strongly inhibited TGF‐*β* signalling by binding to *α*_v_ *β*_3_ integrin and reduced extracellular matrix deposition and liver fibrosis in mice.^17^ In the aspect of cardiac fibrosis, Deng et al considered *MFGE8* as an endogenous negative regulator of pathological cardiac hypertrophy, because after aortic coarctation in *Mfge8*‐knockout (*Mfge8*‐KO) mice, substantial hypertrophic enlargement in cardiomyocytes, systolic dysfunction, and myocardial fibrosis was observed.^13^ In our previous work, MFGE8 suppressed atrial fibrosis via TGF‐β1/Smad2/3 pathway in an integrinβ3‐dependent way and attenuated the vulnerability to atrial fibrillation.^19^ Accordingly, in the present study, immunofluorescence images showed significantly decreased expression of MFGE8 in Vimentin‐labelled fibrosis region in the rat model of TAC‐induced cardiac fibrosis compared with that of the control group (Figure 1B). Moreover, both in the LV and aorta tissues, vascular endothelial cells showed decreased fluorescence intensity of CD31 and increased fluorescence intensity of Vimentin, indicating that ECs underwent EndMT after TAC (Figure 1B,C). Therefore, we hypothesized that MFGE8 might participate in the occurrence and development of cardiac fibrosis by regulating EndMT. As shown in the above experimental results, knock‐down of MFGE8 promoted TFG‐*β*1‐induced EndMT (Figures 3, 4, 5) while administration of rh‐MFGE8 reversed the process (Figure 6), and Smad2/3 pathway and transcription factors such as Snail are critical mediators (Figure 7, Figure S1). In addition, MFGE8 acts as a momentous bridging molecule that binds to integrin receptors, such as *α*_v_ *β*_5_ on phagocytes and phosphatidylserine on dead cells. Nakaya et al found that myofibroblasts that have underwent EndMT secrete MFGE8 to mediate phagocytosis of dead cells and facilitate recovery after MI.^15^ Interestingly, our study demonstrated that in vitro administration of rh‐MFGE8 protects endothelial cells from undergoing EndMT. Such a circulation of MFGE8 and EndMT in endothelial cells and myofibroblasts is likely to be the evidence of the protective role of MFGE8 in heart fibrosis. We can speculate that the administration of rh‐MFGE8 prior to cardiac fibrosis may have clinical implications for inhibiting the progress of cardiac remodelling or even heart failure.

In addition, an interesting phenomenon aroused our thinking. Structurally, the second epidermal growth factor (EGF)‐like domain of the NH2‐terminal of MFGE8 contains an arginine‐glycine‐aspartic acid (RGD) motif, and the COOH terminus of MFGE8 has a factor VIII‐like domain (C2), which enables it to bind to phosphatidylserine on apoptotic cells and *α_v_β_3_*_/_ *_5_* integrins on phagocytic cells as a bridging molecule.^38^, ^39^, ^40^ Jinushi et al found that in the mouse melanoma model, MFGE8 stimulated melanoma cell resistance to apoptosis and triggered EMT by activating Akt pathway and stimulating the production of Snail and Twist transcription factors, thus enhancing tumorigenicity and metastasis ability.^41^ Silvestre et al believed that endogenous MFGE8 specifically promoted vascular endothelial growth factor (VEGF)‐dependent Akt signalling to promote angiogenesis.^42^ In these two pathological environments, endogenous MFGE8, as a key cofactor of VEGF, activates Akt pathway to stimulate angiogenesis to promote tumour development or improve cardiac remodelling after myocardial infarction. In our study, MFGE8 knock‐down does inhibit angiogenesis under the existence of TGF‐*β*1 (Figure 5A,B). Nonetheless, another study showed that MFGE8 alleviated myocardial hypertrophy through inhibition of Akt pathway.^13^ A previous study by Atabai et al found that MFGE8‐mediated collagen internalization could reduce the severity of murine tissue fibrosis in a manner independent of RGD‐binding integrins.^18^ In our current study, MFGE8 regulated EndMT by inhibiting the Smad2/3 pathway and reducing the production of EndMT‐related transcription factors. These results indicated the multifunctional roles of MFGE8 in different models of the diseases. According to our analysis, MFGE8 does not affect body development and function under normal physiological conditions but can play specific role through its multiple domains at different pathway levels when different pathological pressures occur, such as inflammation, tumour and ischaemia. As shown in our results, the effects of *MFGE8* knock‐down or rh‐MFGE8 administration on EndMT‐related proteins in the absence of TGF‐*β*1 stimulation were not statistically significant compared with the control group. We believe that in order to accurately explore the mechanism of MFGE8 in CF and HF, it is significant to construct cardiac‐specific *Mfge8* conditional knockout experimental animals in our subsequent experiments.

In conclusion, the above findings indicate that MFGE8 suppresses the pathogenesis of TGF‐*β*1‐induced EndMT by inhibiting the Smad2/3 signalling pathway and regulating EndMT‐related transcription factors. Our research demonstrates a novel molecular mechanism of the function of MFGE8 in EndMT and further proves the use of MFGE8 as a potential circulating biomarker or therapeutic target for diagnosis and mitigation of CF and CF‐related heart failure. On the other hand, our results still have limitations. Other pathways such as Notch signalling may also be regulated by MFGE8, and we hope to create conditional cardiac knockout rats to get the results that are more persuasive in subsequent studies.

## CONFLICT OF INTEREST

The authors have read and approved the final manuscript for submission and declare no conflict of interest.

## AUTHOR CONTRIBUTIONS

**Bo Wang:** Data curation (equal); investigation (equal); methodology (supporting); writing‐original draft (supporting). **Zhuowang Ge:** Data curation (equal); investigation (equal). **Yan Wu:** Data curation (equal); investigation (equal). **Yafang Zha:** Methodology (supporting). **Xuan Zhang:** Methodology (supporting). **Yexiang Yan:** Methodology (supporting). **Yu quan Xie:** Methodology (lead); project administration (lead); supervision (lead); validation (lead); writing‐original draft (lead); writing‐review and editing (lead).

## Supporting information

Fig S1

## Data Availability

Data supporting the findings of this study are included in the article.
