# Supplementary figures and images for: MFGE8 is down‐regulated in cardiac fibrosis and attenuates endothelial‐mesenchymal transition through Smad2/3‐Snail signalling pathway
Source: J Cell Mol Med. 2020 Sep 17;24(21):12799–812. doi: 10.1111/jcmm.15871 (PMC7686985; doi:10.1111/jcmm.15871)

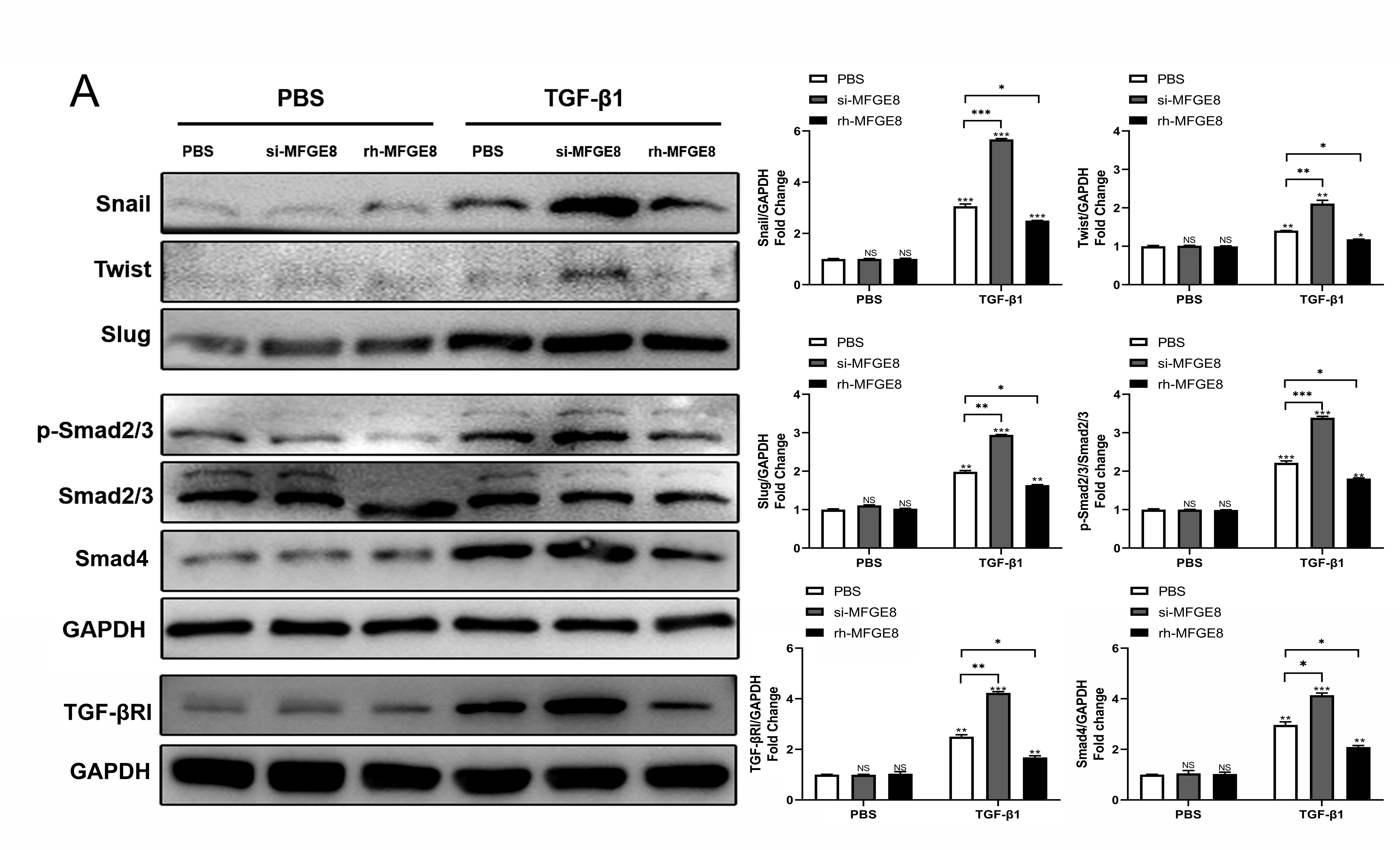

Supplement: Supplementary file 1 — Fig S1 [file JCMM-24-12799-s001.tif]
